# Acknowledgement to Reviewers of *Medical Sciences* in 2018

**DOI:** 10.3390/medsci7010008

**Published:** 2019-01-11

**Authors:** Medical Sciences Editorial Office

**Affiliations:** MDPI, St. Alban-Anlage 66, 4052 Basel, Switzerland

Rigorous peer-review is the corner-stone of high-quality academic publishing. The editorial team greatly appreciates the reviewers who contributed their knowledge and expertise to the journal’s editorial process over the past 12 months. In 2018, a total of 114 papers were published in the journal, with a median time to first decision of 21 days and a median time to publication of 47 days. The editors would like to express their sincere gratitude to the following reviewers for their cooperation and dedication in 2018:

Abdeltawab NourtanBeninati Prof. SimoneAbuzaid AhmedBenitez Arciniega AlejandraAdamantia LiapikouBennett James P.Aeddula Narothama ReddyBerckmoes Lidewyde H.Afolayan A. J.Bergamini CarloAlcázar BernardinoBernardini GiuliaAllen HerbertBessone FernandoAlsharairi NaserBhave DevayaniAmthor HelgeBidoli EttoreAnsa BenjaminBircher JohannesAntonello NicoliniBittner Edward A.Anuurad ErdembilegBizzaro DeboraAranki DanielBjörkman AndersArcher NormBoix EsterAshish PathakBolaris MichaelAspalter RosaBongaerts BrendaAucoin MoniqueBouchecareilh MarionBachmann AndreBreen Ronan A.M.C.Bahnson EdwardBrevern AlexandreBalmaks ReinisBrisbois ElizaebthBarnes Jarrod W.Brouwer MirandaBarnes Neil C.Bruisten SylviaBarratt Shaneybuechler christaBarrott JaredBuendía Roldán IvetteBasso ManuelaBueno MartaBauer MoisesBurger MichaelButler MarkD'Eletto ManuelaCabrera CarlosDelorme VincentCaldron Paul H.Dembiński ArturCalle Rubio MyriamDkhar Hedwin KitdorlangCamiciottoli GiannaDomingo-Calap PilarCampoy SusanaD'Onofrio GraziaCaneiras CátiaDu XingrongCapoccia MassimoDženkevičiūtė VilmaCardenas Zuniga RobertoEdeas MarvinCastro PedroEdmonds MichaelCavallini GiorgioEfird JimmyCavoretto PaoloEinbinder YaelCeccato AdriánEkúndayò Olúgbémiga T.Celano Jorcin LauraEl Aidy SaharCenik BasarErben Mauricio F.Ceranowicz PiotrErrichiello EdoardoCeroni DimitriEscario JoséChakraborty RajaFagiolini AndreaChang Jer-MingFernando De Rezende LuizChatterjee IshitaFinegold Sydney M.Chauhan SmitFischer MatthiasChen Yi-MingForbes LouisaChhabra Harvinder SinghFortes ZuleicaClausen JohannesFoty Ramsey A.Cleveland John L.Franco ElisabettaCoelho CamilaFrank SašaColuccia MauroFrascoli MichelaCommon JohnFregoso TomasConduit RussellFunder Jonas AmstrupCook Alonzo D.Gabazza Esteban C.Coussons PeterGamero Ana M.Cox LauraGani FedericaCox Linda S.García Rivero Juan LuisCrisafulli ErnestoGargaro MarcoCruz FernandaGarraud OlivierCunningham PatrickGarssen BertCurry ScottGentile VittorioCurtin PaulGerner Eugene W.Dakhlallah DuaaGerratana LorenzoDe Brum Vieira PatríciaGianella CamilaDe Miguel-Díez JavierGilmour SusanDe Nardo PasqualeGirardis MassimoDe Paco Matallana CatalinaGiron Maria CeciliaGizzi AlessioJohnson KyleGniadek AgnieszkaJones Barbara E.Goel PeeyushJones MalcolmGomez De Agüero MercedesJones RobinGoto YoshiyukiJu XinshengGray AndrewKamijo-Ikemori AtsukoGray BenKanaseki TakayukiGreenberg Charles S.Kashiwagi ShinichiroGrieco DomenicoKędzierska EwaGris Jean ChristopheKelchtermans HildeGrohmann UrsulaKent BrianneGromov IrinaKern JanetGualillo OresteKhatir Dinah S.Gummerson NigelKieffer NicolasHadžović-Džuvo AlmiraKim Soo-YoulHagan ChristopherKocot JoannaHagen Ralf MatthiasKoltz Michael T.Han JayoungKomori TeruhisaHenry Brandon MichaelKonstantinidis Theocharis G.Hildreth BlakeKorfei MartinaHorhat Florin GeorgeKornguth Steven E.Hsieh Yi-ChenKrishna JyotiHsu LewisKumar SunilHuang YiKuroda KeijiHui-Ching WuLa Favor Justin D.Hulten Kristina G.Labarinas SoniaIacono DiegoLa-Beck Ninh (Irene)Ientile RiccardoLanbeck PeterIgarashi KazueiLebensztejn Dariusz M.Inamura KentaroLedo AnaIrwin David C.Lee Chi Yan VivianIto NaokiLee SunjaeIvanenko AnnaLeong PaulIvanenko AnnaLi Shengwen CalvinJabłonowski ZbigniewLi WeijuanJardini Maria Aparecida NevesLi ZhillingJat Parmjit S.Lin Chih-LiJaved MahmoodLin Ro-TingJena Prasant KumarLitofsky Norman ScottJenkins IanLleo De Nalda AnaJin JiefuLohner KarlJo Helen E.Lombardi AngelaJohnson Amy K.Lonardo AmedeoŁoniewski IgorNakamura HitomiLosurdo GiuseppeNana Andre W.Luchetti MicheleNandi SaikatLumbreras HortensiaNaumann David N.Luppi FabrizioNikiforov MikhailMachado Mariana V.Nosetti LuanaMacín Stella MarisNowicka GrażynaMackay AlexNowotarski ShannonMagori KrisztianOhshimo ShinichiroMalan ValerieOkada TakashiMalik DanishO'Neill Thomas J.Malojcic BrankoOrbach DanielMárquez-Martín EduardoOredsson StinaMasalova Olga V.Origanti SofiaMascherini GabrieleOsier NicoleMassie CliffordOuburg SanderMastorci FrancescaPace FabioMather CareyPajkrt EvaMatsufuji SenyaPalini SimoneMcIntosh E. David G.Pan XiMedina RoxanaParashar DeepakMeng Yuan XiangParfrey HelenMenzel StephanPark Jun-BeomMesquita JoãoPark Myung HeeMiles Wayne OwenPark Myung HeeMoghadamyeghaneh ZhobinPatwari PallaviMonji AkiraPavord Ian D.Monni GiovanniPawelec GrahamMoores CarlyPedersen Kim B.Morales Suárez-Varela María M.Penke BotondMorese RosalbaPennock NathanMorioka ShoPerales AlfredoMoriwaki KentaPergament EugeneMortensen EricPerticone FrancescoMoser OthmarPeters EvaMotawi Tarek K.Pistelli FrancescoMotherway Mary O’connellPiu SahaMoturi SricharanPlate ManuelaMuñoz-López FranciscoPlummer VirginiaMurai NoriyukiPohl GerdaMurray Thomas ScotPolo EsterMyles Ian A.Potus FrançoisNaber Kurt G.Poudel BarunPowell HeatherShah Kevin S.Prêle CeciliaShahi ShaileshPrieto-Lloret JesusShander AryehProudfoot Amanda E. I.Shantz LisaPunganuru Surendra ReddySheu Gwo-TarngRaknes GuttormShi FengRamos VictoriaShiao S. Pamela K.Ramtekkar UjjwalSil PayelRamtekkar UjjwalSingh VishalRanque BrigitteSivapalan PradeeshRathinasabapathy AnandharajanSivaprakasam SathishRawstorn Jonathan C.Skatchkov SergueiReimann Regina R.Soares RaquelReinhardt ChristophSosna JustynaRelia SachinSousa NelsonRezaei AsgharSrivenugopal KalkunteRhee Chae-SeoSteves Claire J.Riccio PaoloStorm HanneRodrigues Celia F.Sundi DebasishRodriguez-Revenga LaiaSzondy ZsuzsaRolfo AlessandroTabolacci ClaudioRoselli MariannaTao JianningRossi ElenaTarantino GiovanniRovers JohnTauler PedroRuddraraju Kasi ViswanatharajuTeschke RolfRuiz-Pérez María VictoriaThomas Bolaji N.Rupérez Azahara IrisThomas T.J.Safoa Martin K.Ticconi CarloSaha AchintoTomar DhanendraSahay BikashTorrado EgídioSanduzzi AlessandroTraves SuzanneSankavaram KavithaTrosman IrinaSapey ElizabethTsilioni IreneSavoia PaolaTuaillon EdouardSawai HirofumiTurnbull DavidSchneider MargueriteTvedt Tor Henrik AndersonSchweiger MartinUrban JillScotti MarcusVan Boven Job FMSecchi Francescovan Dam Anne-MarieSeckeler Michael D.Van Der Velde EnnoSedic MirelaVan Dijk JitseSellarés JacobVan Laar TriciaSerafini GianlucaVan Tine Brian AndrewVathipadiekal VinodWilson BrianVenkatesan ThiagarajanWilson ErinVercellotti GregoryWingfield ThomasVerderio ElisabettaXiang DongxiVerschoor ChrisXU QINZIViasus DiegoYeh Te-HueiVilcinskas AndreasZachou KalliopiVinnedge Lisa PrivetteZagon Ian S.Volicer LadislavZahedi KamyarWang Peng-HuiZakaria DalaWattchow DavidZarogoulidis PaulWebster BonnieZhang KeWeiss ShellyZheng PanWhitfield Kyle Y.Ziejewski MariuszWillis Rohan


